# Percutaneous or mini-invasive surgical radiofrequency re-ablation of atrial fibrillation: Impact on atrial function and echocardiographic predictors of short and long-term success

**DOI:** 10.3389/fcvm.2022.928090

**Published:** 2022-10-31

**Authors:** Sílvia Montserrat, Luigi Gabrielli, Roger Borràs, Enric Cascos, Manel Castellá, Laura Sanchis, Bart Bijnens, Lluís Mont, Marta Sitges

**Affiliations:** ^1^Hospital Clínic, IDIBAPS, Institut Clinic Cardiovascular (ICCV), Centro de Investigación Biomédica en Red (CIBER), Cardiovascular Instituto de Salud Carlos III, University of Barcelona, Barcelona, Spain; ^2^Department of Cardiology, Consorci Hospitalari de Vic, Universitat Central de Catalunya, Barcelona, Spain; ^3^Advanced Center for Chronic Diseases, Escuela de Medicina, Pontificia Universidad Católica de Chile, Santiago, Chile; ^4^Department of Engineering, Institució Catalana de Recerca i Estudis Avançats (ICREA)—Universitat Pompeu Fabra, Barcelona, Spain

**Keywords:** LA function after catheter/surgical re-AF ablation atrial fibrillation, atrial function, echocardiography, catheter ablation, surgical ablation, strain and strain rate

## Abstract

**Objectives:**

The aim of this study was to compare percutaneous catheter ablation vs. minimally invasive surgical ablation, evaluating the impact of repeated ablation on atrial function, and evaluating predictors of atrial fibrillation (AF) recurrence.

**Background:**

When AF ablation fails, re-ablations are required in up to 40% of patients to treat recurrent arrhythmia; surgical ablation is more effective than catheter ablation.

**Methods:**

Thirty-two patients with failed prior catheter ablation and referred for a second ablation (18 catheter and 14 surgical) were included in a descriptive observational study. Left atrial volumes, strain, and strain rate were measured with 2D speckle tracking echocardiography at baseline and 6 months after the procedures to assess left atrial functions. Patients received up to 1 year of clinical and Holter follow-up.

**Results:**

At the 12-month follow-up, catheter ablation was effective in 56% and surgical ablation in 72% of patients (OR 2 (CI 0.45–8.84), *p* 0.36). Left atrial booster function was similar in all patients, but left atrial reservoir function was more impaired in those patients who underwent surgical ablation. Left atrial booster function was predictive of arrhythmia recurrence after both catheter and surgical ablation: late diastolic strain rate (LASRa) cut-off ≤ -0.89 s^–1^ (sensitivity 88%, specificity 70%, AUC 0.82) and ≤ -0.85 s^–1^ (sensitivity 60%, specificity 100%, AUC 0.82), respectively.

**Conclusion:**

Surgical ablation has a more negative impact on LA reservoir function despite being slightly more effective in arrhythmia suppression. LA booster function is not significantly impaired by either procedure. LA booster function predicts arrhythmia elimination after a re-ablation (catheter or surgical).

## Introduction

After an initial radiofrequency catheter ablation, both percutaneous catheter (CA) and minimally invasive surgical (SA) ablation are accepted therapeutic options for the treatment of patients with recurrent atrial fibrillation (AF) (1). A second procedure is required in up to 40% of patients undergoing AF ablation. Indeed, the efficacy of a second ablation in patients with AF recurrence after a first ablation is reported to be approximately 58%, while antiarrhythmic drugs are only effective in 27% of these patients (2). The efficacy of AF ablation increases with the number of procedures: arrhythmia-free survival rate at 5 years after CA is reported to be around 29% with one procedure and can increase to 63% with two or more procedures (3). In addition, AF ablation with minimally invasive surgery has shown excellent short- and mid-term results. Data suggests that SA more effectively eliminates the arrhythmia in patients with recurrent AF after a failed CA, although CA is associated with fewer complications (4).

Nonetheless, the impact of such procedures on left atrial (LA) function is not well known. Additionally, guidelines on candidate selection for either procedure have not been well established due to lack of data regarding potential factors predicting procedural success, particularly for SA. Despite evidence that LA size, function and fibrosis (as surrogates of the underlying atrial AF substrate) are all related to the success of a first AF ablation procedure, no studies have specifically compared these parameters in the setting of redo CA and SA procedures.

Therefore, the aim of this study was to evaluate, first, the impact of redo CA and SA on atrial function and second, to analyze the potential role of LA size and function in selecting the best candidates for each technique, in order to optimize the rate of long-term success in eliminating the arrhythmia.

## Materials and methods

### Study population and study protocol

A descriptive observational study analyzed two cohorts of patients undergoing SA or CA. The study included 32 patients (81% men, 53 ± 7 years old) with symptomatic, antiarrhythmic, drug-refractory AF treated with at least one antiarrhythmic drug and with a failed prior percutaneous ablation. All patients had symptoms before the intervention: palpitations, shortness of breath, and anxiety for the possibility of a symptomatic AF episode. Table 1 shows the clinical [age, hypertension, BSA, paroxysmal or persistent AF, AF duration (84–72 months), and antiarrhythmic drugs] (2) and the echocardiographic characteristics of the studied population before CA and SA.

**TABLE 1:** Baseline clinical and echocardiographic parameters.

	Catheter ablation (*n* = 18)	Surgical ablation (*n* = 14)	*p*-value
Age (years)	52[49–59]	55 [47–57]	0.94
Hypertension (%)	11p (61%)	3p (21%)	0.04
Body surface area (BSA) (m^2^)	1.99 [1.92–2.07]	2.15 [1.81–2.17]	0.50
Antiarrhythmic drugs (number)	2 [1–2]	2 [1.5–2]	0.37
AF duration (month)	84 [36–114]	72 [48–133]	0.69
Paroxysmal AF *n* (%)	10 (56%)	11 (79%)	0.27
LV hypertrophy *n* (%)	6 (33%)	3 (21%)	0.69
LV EF (%)	60 [60–60]	60 [59–65]	0.67
LV end-diastolic diameter (mm)	55 [49–57]	52 [50–55]	0.19
LV end-systolic diameter (mm)	33 [31–36]	32 [27–34]	0.34
LA anteroposterior diameter (mm)	41 [34–44]	40 [37–42]	0.77
LA anteroposterior diameter/BSA (mm/m^2^)	21 [18–22]	20 [18–21]	0.45
LA maximum volume (ml)	68 [50–78]	52 [46–61]	0.25
LA maximum volume/BSA (ml/m^2^)	36 [22–42]	27 [24–35]	0.87
LASs_l_ **(%)**	17 [12–19]	15 [13–18]	0.63
LASRs_l_ (s^–1^)	0.71 [0.67–1.10]	0.90 [0.72–1.40]	0.89
LASRe_l_ (s^–1^)	−1.19 [−1.58/−1.03]	−1.20 [−1.65/0.90]	0.97
LASRa_l_ (s^–1^)	−0.80 [−1.07/−0.59]	−0.80 [−1.02/−0.52]	0.73

Data expressed as number of patients (*n*) and percentage or median and interquartile range, as appropriate. AF, atrial fibrillation; BSA, body surface area; EF, ejection fraction; LA, left atrium; LV, left ventricle; Ss_*l*_, systolic strain; SRa_*l*_, late diastolic strain rate, SRe_*l*_, early diastolic strain rate; SRs_*l*_, systolic strain rate.

Eighteen participants underwent repeated CA while fourteen subjects underwent a redo SA. We excluded patients with significant valve disease (more than mild regurgitation), severe ventricular hypertrophy (wall thickness in end-diastole > 14 mm), or major ventricular dysfunction (left ventricular ejection fraction ≤ 35%) and patients in AF during echocardiography at follow-up. A repeated procedure was indicated when patients persisted with symptomatic AF after a blanking period of 3 months after the first ablation, despite appropriate pharmacologic treatment. In all patients, two transthoracic echocardiographies were performed before the redo procedure, either CA or SA, and at the 6-month follow-up. All patients were in sinus rhythm when the echocardiogram was registered The Ethics Committee of our institution approved the study and all patients signed an informed consent.

### Percutaneous radiofrequency catheter ablation

The LA and pulmonary veins were explored using a transseptal approach. A 3-dimensional map was constructed using an electroanatomic mapping system (CARTO^®^, Biosense-Webster, Diamond Bar, CA, USA) to support the creation and validation of radiofrequency lesions. Continuous radiofrequency lesions surrounding ipsilateral were delivered as previously described (5, 6). In patients with persistent AF, additional radiofrequency applications were made along the LA roof (between superior pulmonary veins), LA posterior wall, and mitral isthmus (between inferior pulmonary veins) at the discretion of the operator. Radiofrequency was delivered through an irrigated-tip thermocouple-equipped catheter, using a target temperature of 45°C at 40 W. The endpoint was a reduction of local electrogram to < 0.15 mV and the establishment of a bidirectional conduction block between the LA and pulmonary veins (6).

### Surgical ablation

Patients were treated with a minimally invasive surgery protocol (4) using video-assisted thoracoscopy, under general anesthesia. Pulmonary vein isolation was performed from the epicardial side with a bipolar RF ablation clamp (Nasdaq:ATC, AtriCure, Inc., Ohio, USA). At least 2 overlapping applications around each of the ipsilateral veins were performed, and isolation was confirmed by the absence of pulmonary vein potentials and exit block during pacing. Ablation of ganglionic plexi was also performed, with an additional application in the interatrial Waterston groove on the right side, while Marshal’s ligament was cut on the left side. In all patients, the LA appendage was excluded by stapling and then cutting.

### Clinical follow-up

After CA and SA, all patients continued oral anticoagulation to maintain an international normalized ratio between 2.0 and 3.0 for a minimum of 3 months; therapy was continued at the discretion of the treating cardiologist on the basis of the CHA2DS2-VASc score (1). Previous antiarrhythmic therapy was maintained for at least 1 month and then discontinued if there were no arrhythmia recurrences 3 months after ablation, except in two patients previously prescribed beta-blockers due to ischemic heart disease and ventricular extrasystolia. Clinical follow-up consisted of outpatient check-ups with serial ECG ambulatory monitoring to evaluate the recurrence and frequency of any potential arrhythmia. All patients underwent a 7-day continuous ECG recording to detect asymptomatic AF episodes at 6 and 12 month follow up. In symptomatic patients but with sinus rhythm in the ECG, ECG monitoring was limited to 48 h.

The ablation procedure was considered successful if no recurrences of atrial tachycardia lasting > 30 s were present during follow-up, after a blanking period of 3 months (1). Also, clinical improvement, defined as fewer clinical palpitations and hospitalizations due to arrhythmia, was evaluated.

### Echocardiography

All patients underwent transthoracic and transesophageal echocardiography prior to the CA or SA procedure, including conventional 2-dimensional echocardiography to detect predictors of success for CA and SA. Echocardiographic follow-up was performed at 6 months to detect the effect of CA and SA. All images were obtained using the IE33 Philips ultrasound system (Philips, Andover, MA, USA), digitally stored, and transferred to a workstation for off-line analysis using dedicated software (Qlab, version 7.1, Philips Medical Systems).

**FIGURE 1:**
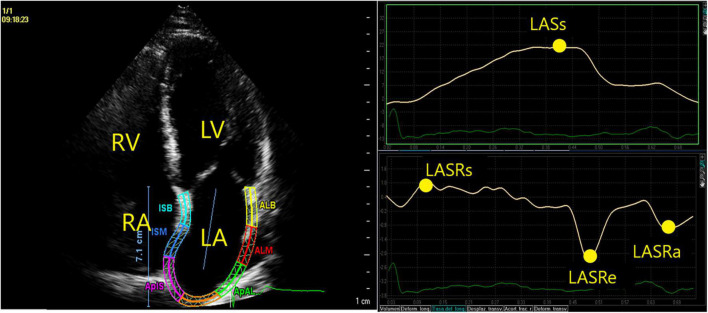
Endocardial border was automatically traced with speckle tracking. LASs_l_, representing LA reservoir function, was identified as the peak positive strain value during LV systole. In the LA strain rate curve, we identified the peak positive strain rate (LASRs_l_) at the beginning of LV systole, the peak negative strain rate during early diastolic (LASRe_l_) strain rate, representing conduction function and late diastolic (LASRa_l_) strain rate after the P-wave on the electrocardiogram, representing LA active contraction.

The LA anteroposterior diameter and left ventricular dimensions were measured in the long parasternal axis; left ventricular ejection fraction was determined using the biplane Simpson method using biplane apical views. LA volumes were measured *via* the disc summation method from apical 4-chamber views, in LA and right atrium. LA function was assessed using myocardial deformation imaging derived from 2D speckle tracking (7, 8) by offline analysis of standard apical 4-chamber views. Special care was taken at the time of image acquisition to focus on the LA and to have a frame rate in the range of 50–70 fps. After selecting 3 points (8) (the inferior part of the LA septum, the inferior part of the LA lateral wall, and the LA roof), an endocardial border trace was generated by the software (Figure 1). The reference point for the initiation of 2D speckle tracking is QRS. The LA strain and strain rate traces were calculated and depicted. The left atrium was divided into 7 segments. The strain and strain rate curves were measured for each segment and the results represent the average value. From the average strain curves, myocardial LA longitudinal systolic strain (LASs _*l*_) was calculated, representing LA reservoir function. LA systolic strain rate (LASRs _*l*_) also represents LA reservoir function; early diastolic strain rate (LASRe _*l*_) represents conduit function; and late diastolic strain rate (LASRa _*l*_) represents LA booster function (Figure 1).

### Statistical analysis

Data is reported as median and interquartile range. Continuous variables were tested by the U Mann-Whitney test, and paired data by Wilcoxon analysis where appropriate. Categorical variables are presented in proportions, and were compared *via* the Fisher exact test. We used ROC methodology to evaluate the optimal cut-off value for predicting recurrence in our sample. Classification trees (9) were constructed in order to select the best LA parameters for predicting ablation success (AF recurrence or non-recurrence). A *P*-value ≤ 0.05 was considered statistically significant. The Lin concordance coefficient was calculated to study the reproducibility of the measured variables (8). Statistical analysis was performed using R software for Windows version 3.1.1 (R project for Statistical Computing; Vienna, Austria).

## Results

### Baseline characteristics and clinical follow-up at 1 year

Table 1 shows the clinical and echocardiographic characteristics of the population studied pre-CA and SA. There were no significant echocardiographic differences between groups undergoing CA or SA, including LA size and function parameters. Hypertension was more frequent in CA patients compared with patients undergoing SA. At the 1-year follow-up, SA was slightly more effective, achieving success in 72% of patients compared with 56% of CA patients [OR 2 (CI 95% 0.45–8.84), *p* = 0.36].

Of the SA failures, 4/14 patients (28%) occurred before 6 months follow-up without any other recurrences between 6 and 12 month follow. Of the CA failures, 4/18 patients (22%) occurred before 6 months and increased to 8/18 patients (44%) before 12-month visits. Clinical improvement, defined as fewer clinical palpitations and hospitalizations due to arrhythmia, was observed in 16 of 18 (89%) patients from the CA group and in 13 of 14 (93%) from the SA group (*p* = 0.99).

### Impact of successful repeated atrial ablation on LA function at 6 months

**FIGURE 2:**
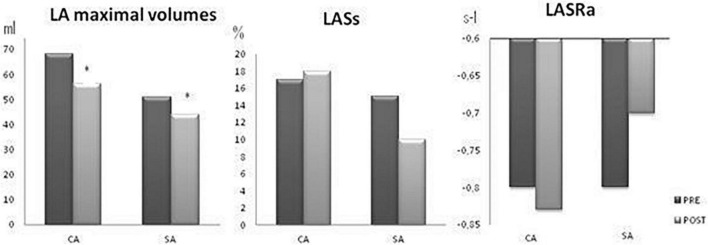
Effect of catheter (CA) and surgical ablation (SA) on LA size (LA 2D maximum volume) and LA reservoir (LASs_l_) and LA booster function (LASRa_l_). PRE, pre-ablation (black bars) and POST, post-ablation (gray bars). CA, Catheter ablation; SA, surgical ablation. *p < 0.05 POST vs. PRE.

Figure 2 shows the effect of CA and SA on LA size and function [reservoir (LASs_l_) and booster LA functions (LASRa_l_)] as studied with deformation imaging of the LA wall (pre- and 6 months after ablation). Both procedures reduced the LA maximum volumes, but did not significantly impair LASs_l_ or LASRa_l_. Comparison between CA and SA only showed a difference in the reservoir function effect. First, LA size showed a similar decrease after both procedures: in patients undergoing CA, maximum LA volume decreased by a median Δ -12 [-2/-21] ml and, for SA, this decrease was Δ -8 [-1/-13] ml (*p* = 0.59). Second, LA reservoir function was more impaired after SA [LASs_l_ decreased by Δ -4.65 (-0.02/-9)%], than after CA [LASs_l_ increased by Δ + 0.75% (-1.1/ + 2.8) (*p* 0.04)]. Third, LA booster function was similarly affected in patients undergoing SA (LASRa_l_ decreased by Δ -0.03 (-0.55/ + 0.29)s^–1^] and in those treated with CA [LASRa_l_ decreased by Δ -0.10^–1^ (-0.23/ + 0.20)]s^–1^ (*p* = 0.83).

**FIGURE 3:**
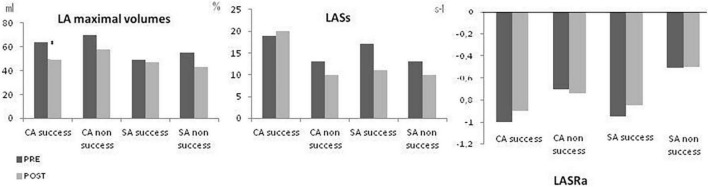
Effect of catheter (CA) and surgical ablation (SA) on LA size (LA maximum volume), LA reservoir function (LASs_l_) and LA booster function (LASRa_l_) depending of the success of the procedure in eliminating AF. PRE: pre-ablation and POST: post-ablation. CA, Catheter ablation; SA, surgical ablation. *p < 0.05 PRE vs. POST.

Figure 3 shows the effect of CA and SA on LA size and function, according to the success of the procedure in eliminating the arrhythmia. LA size was reduced in all subgroups (CA/SA, successful/non-successful) but the reduction was significant only in patients with successful CA. Compared to preprocedure imaging, LA booster function showed no significant changes and was stable after both CA and SA, independently of the success of the ablation. LA reservoir function slightly improved after successful CA (*p* = 0.47) and slightly decreased after non-successful CA (*p* = 0.09). However, LA reservoir function slightly worsened after SA whether successful (*p* = 0.26) or not (*p* = 0.07).

### Predictors of success after repeated atrial ablation

Tables 2, 3 show the baseline characteristics of patients undergoing CA and SA, respectively, according to the success of the procedure. Despite similar LA size, LA reservoir and booster function were significantly more preserved, as shown by higher LASs_l_, LASRs_l_ and LASRa_l_, in patients undergoing a successful redo CA (Table 2). A similar trend was observed in the SA group, with better LA booster function in patients with a successful SA (Table 3).

**TABLE 2:** Baseline characteristics of patients undergoing CA according to the success of the procedure.

	Successful CA (*n* = 10)	Non-successful CA (*n* = 8)	*p-*value
Age (years)	53 [50–56]	52 [46–69]	0.86
Hypertension *n* (%)	4 (40%)	7 (88%)	0.07
AF duration (months)	66 [24–126]	84 [36–96]	0.85
Paroxysmal AF *n* (%)	7 (70%)	3 (38%)	0.34
Antiarrhythmic drugs (number)	1.5[1–2]	2 [1–2]	0.61
LV hypertrophy *n* (%)	4 (40%)	2 (25%)	0.64
LV EF (%)	60 [59–62]	60 [60–60]	0.87
LA anteroposterior diameter (mm)	41 [32–44]	42 [36–46]	0.48
LA AP diameter/BSA (mm/m^2^)	20 [17–22]	22 [18–23]	0.49
LA maximum volume (ml)	64 [36–92]	70 [56–73]	0.79
LA maximum volume/BSA (ml/m^2^)	36 [17–44]	35 [29–37]	0.87
LASs_l_ (%)	18 [15–22]	13 [7–17]	**0.03**
LASRs_l_ (s^–1^)	0.98 [0.83–1.17]	0.73 [0.58–0.95]	**0.02**
LASRe_l_ (s^–1^)	−1.20 [−1.53/−1.01]	−1.18 [−1.75/−1.02]	0.86
LASRa_l_ (s^–1^)	−1.01 [−1.42/−0.78]	−0.69 [−0.80/−0.54]	**0.02**
RA maximum volume (ml)	30 [19–54]	43 [42–51]	0.43
A wave (cm/s)	51 [42–62]	43 [42–51]	0.53

Data expressed as number of patients (*n*) and percentage or median and interquartile range as appropriate. Bold values indicate statistical significance. AF, atrial fibrillation; AP: anteroposterior; A wave, late diastolic transmitral wave; BSA, body surface area; EF, ejection fraction; LA, left atrium; LV, left ventricle; RA, right atrium; Ss_*l*_, systolic strain; SRa_*l*_, late diastolic strain rate, SRe_*l*_, early diastolic strain rate; SRs_*l*_, systolic strain rate.

**TABLE 3:** Baseline characteristics of patients undergoing SA according to the success of the procedure.

	Successful SA (*n* = 10)	Non-successful SA (*n* = 4)	*p*-value
Age (years)	50 [50–57] 8 (80%)	47 [38–58]3 (75%)	0.26
Hypertension *n* (%)	2 (20%)	1 (25%)	1
AF duration (months)	72 [48–120]	72 [48–150]	0.92
Paroxysmal AF *n* (%)	7 (70%)	4 (100%)	0.51
Antiarrhythmic drugs (number)	2 [1.25–2]	2 [2–2]	0.60
LV hypertrophy *n* (%)	2 (20%)	1 (25%)	1
LV EF (%)	63 [60–65]	58 [40–60]	0.05
LA anteroposterior diameter (mm)	40 [37–42]	39 [37–44]	0.88
LA AP diameter/BSA (mm/m^2^)	20 [18–21]	20 [18–24]	1
LA maximum volume (ml)	49 [44–61]	55 [49–71]	0.36
LA maximum volume/BSA (ml/m^2^)	25 [23–35]	31 [23–36]	0.81
LASs_l_ (%)	17 [13–20]	13 [13–15]	0.22
LASRs_l_ (s^–1^)	0.95 [0.79–1.77]	0.70 [0.57–0.92]	0.17
LASRe_l_ (s^–1^)	−1.15 [−2.02/−0.87)]	−1.30 [−1.92/−1.13]	0.39
LASRa_l_ (s^–1^)	−0.95 [−1.47/−0.52)]	−0.50 [−0.73/−0.39]	0.04
RA maximum volume (ml)	49 [41–59]	53 [50–75]	0.41
A wave (cm/s)	58 [41–66]	37 [27–43]	0.05

Data expressed as number of patients (*n*) and percentage or median and interquartile range as appropriate. AF, atrial fibrillation; AP, anteroposterior; A wave, late diastolic transmitral wave; BSA, body surface area; EF, ejection fraction; LA, left atrium; LV, left ventricle; RA, right atrium; Ss_*l*_, systolic strain; SRa_*l*_, late diastolic strain rate, SRe_*l*_, early diastolic strain rate; SRs_*l*_, systolic strain rate.

**FIGURE 4:**
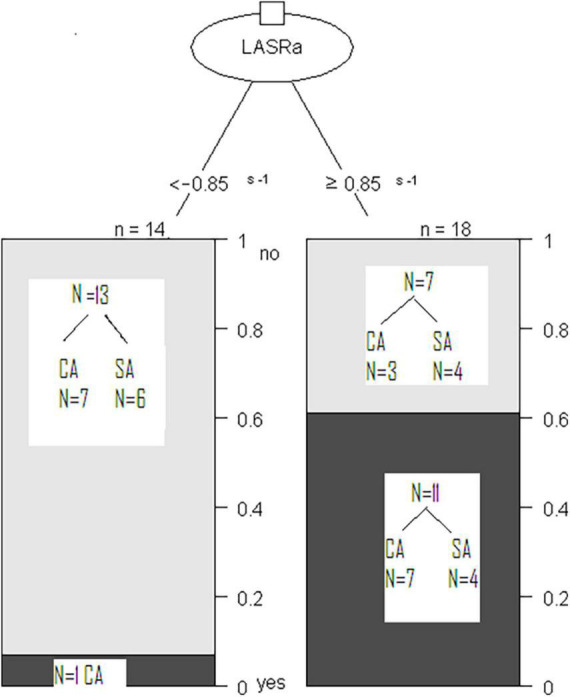
Classification Tree Model to predict arrhythmia elimination with repeated procedures (CA or SA) based on LA contractile function as determined by LASRa_l_. Patients with non-recurrence of AF are represented by gray bars **(Top)** and those with recurrence of AF by black bars **(Bottom).**

The LA booster function (LARSa _*l*_) predicted the success of second procedures, whether CA or SA. The patients with LASRa_l_ < -0.85 s^–1^, 93% (13/14) had no AF recurrence (54% in the CA group, 7 patients; 46% in the SA group, 6 patients). Among patients with impaired LA booster function (LASRa_l_ ≥ -0.85 s^–1^), the arrhythmia was eliminated after the second procedure in 39% (7/18): 3 CA patients and 4 SA patients. The remaining 61% (11/18) had a non-successful second procedure [64% (7/11) CA patients and 36% (4/11) SA patients] (Figure 4).

In the ROC (Receiver operating characteristic) analysis, LA booster function (LASRa_l_ cut-off ≤ -0.89 s^–1^) predicted arrhythmia elimination after CA (sensitivity 88%, specificity 70%, AUC 0.82). LA reservoir function also predicted CA success (LASs_l_ cut-off > 15%; sensitivity 71%, specificity 80%, AUC 0.79). Among patients undergoing SA, the LA booster function was the only predictor of ablation success: LASRa_l_ cut-off ≤ -0.85 s^–1^ (sensitivity 60%, specificity 100%, AUC 0.82).

Of the CA failures, 4/18 patients (22%) occurred before 6 months and increased to 8/18 patients (44%) before 12-month visits.” LASs (not LA size) post CA at 6 months predicts the recurrence at 12 month (OR 0.64, IC 0.44–0.92; *p* 0.02).

### Reproducibility study

**FIGURE 5:**
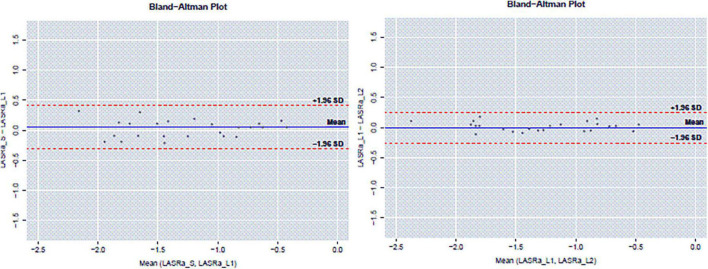
Reproducibility of LASRa_l_ measurements: inter-observer Lin concordance and intra-observer concordance.

The reproducibility of LASRa_l_ measurements was excellent: Both inter-observer and intra-observer Lin concordance was 0.97 (Figure 5). The concordance value is classified as Poor (< 0.90), Moderate (0.90–0.95), Substantial (0.95–0.99), and Almost perfect (> 0.99) (9).

## Discussion

The main finding of the study was that LA booster function is preserved after repeated CA and SA, suggesting that repeated ablation is useful for avoiding AF complications and increasing the success of ablation procedures without further harm to LA function induced by ablation lesions and potential induced scarring. In a previous meta-analysis (10), successful CA in AF patients significantly decreased LA size and did not appear to adversely affect LA function, as studied *via* 3D echocardiography and magnetic resonance. This finding was also reported in the subgroup of patients undergoing repeated CA ablation (11). In our study, LA remodeling (reduction of LA size) was observed in both CA and SA.

A previous study also suggested that minimally invasive surgical AF ablation (12) (first and repeated procedures) improves LA function as measured *via* 2D speckle-tracking echocardiography. In our study we evaluated the impact of SA on LA size and function, and compared it with that seen in patients undergoing repeated CA where previous CA had failed. We found that, at the 6-month follow-up, LA reservoir function had decreased more in patients treated with SA than in those receiving repeated CA. The LA reservoir function depends on left ventricular systolic function and LA compliance. In our patients, left ventricular systolic function was preserved, but the impairment of LA compliance was higher in the SA group of patients than in CA patients. This finding might be secondary to several factors: a more extensive ablation, more wall fibrosis due to the epicardial approach, superior transmurality of the bipolar radiofrequency lesion, concomitant ablation of ganglia, and the elimination of LA appendage by SA. All these factors might influence LA compliance, and consequently, LA reservoir function. However, LA booster function was not significantly impaired by ablation after either the CA or SA procedure.

While electrical restoration is mandatory, restoration of LA contraction, and mechanical function is also important after CA and SA. Long-term cardiovascular events could depend on the evolution of LA function in these patients, as they could potentially maintain sinus rhythm after ablation, but with reduced LA function, and consequently, an increased risk of embolism and AF recurrence.

Enlarged LA and impaired ResF (LA ResF [emptying fraction (LAEF) and expansion index (LAEI)]) at 3-month post-ablation for AF are strongly associated with long-term outcomes (cardiac hospitalization for heart failure or acute ischemic events, stroke/TIA, and all-cause death), independent of LV function or cardiac rhythm at follow-up (13).

Indeed, the continuation of the anticoagulation treatment currently depends on the CHA2DS2-VASc score; however, knowing the status of LA function might have therapeutic implications in the future management of these patients. Some studies report that LA strain is reduced in patients with AF, stroke, and low risk CHADS 2 scores (14). Finally, this is important in terms of the strong cardiovascular prognostic implication of LA contractile function in patients with chronic hypertension and diastolic dysfunction (15) frequently underlying AF.

Actually LA function determined by 2D Speckle tracking appears to be a promising technique for diagnosis and therapeutic decision-making (16).

It is a powerful biomarker for adverse events in different cardiovascular diseases. Recent review describes the methodology, benefits, and pitfalls of measuring LA longitudinal strain function by echocardiography (17).

### Predictors of success after repeated atrial ablation

Another finding in our study was that the LA function status predicts the success of a second ablation procedure (CA and SA) in eliminating arrhythmia after 1-year follow-up. LA function measured *via* 3D echocardiography failed to detect predictors of a second CA (18). Some studies have aimed to assess factors related to the success of a second CA. Wójcik et al. (19) studied 42 patients after a second ablation procedure (5 of them undergoing SA) and identified 3 predictors of success: paroxysmal AF, normalized LA area ≥ 10.25, and a high score with a combination of the AF type, LA size, renal insufficiency, presence of metabolic syndrome or cardiomyopathy (ALARMEc score). Similarly, Tang et al. (20) studied patients before undergoing a second AF ablation procedure. Increased anteroposterior LA diameter, measured by M-mode after the first procedure, was the only independent predictor of AF recurrence after the second procedure. Interest in understanding atrial fibrillation substrate is growing as a predictor for the success of repeated procedures of CA (21). Finally, *via* myocardial deformation imaging techniques, we previously demonstrated that LA reservoir function (LASs _*l*_) is the best predictor of AF recurrence described to date. After repeated CA, at the 6-month follow-up, the sensitivity, and specificity were high (LASs_l_ cut-off > 12%, sensitivity 84%, specificity 90%, AUC 0.89) (8). The present study confirms the importance of LA reservoir function in predicting AF recurrence, but extends its prognostic utility up to 12 months after CA (LASs_l_ cut-off > 15%; sensitivity 71%, specificity 80%, AUC 0.79). The LA reservoir function, as measured by myocardial deformation imaging techniques, inversely correlates with fibrosis in the atrial wall, detected using cardiac magnetic resonance (22), and also with fibrosis detected by histology in atrial wall samples extracted during cardiac surgery in patients with severe mitral insufficiency (23). Therefore, LASs_l_ can be considered as a surrogate of atrial wall fibrosis measurement. Finally, the data we present shows the relevance of LA booster function (LASRa_l_) in predicting the success of CA after a 12-month follow-up.

Only one study has addressed the identification of factors that might predict the success of SA after a failed percutaneous ablation (24). In this study, > 1-year persistent AF and non-dilated LA (anteroposterior diameter < 45 mm) were related to the success of SA in eliminating the arrhythmia. In contrast, our present study, which only included patients in sinus rhythm, demonstrated the value of LA booster function, in addition to LA size, in predicting the success of SA.

New LA functional parameters derived from 2D speckle tracking are of great utility in detecting effect and predictors of second AF procedures (CA vs. SA). From the clinical point of view, patients treated with CA have a less impaired LA reservoir function than patients treated with SA, thus, CA seems to be the best option for patients with more preserved LA booster and reservoir function. However, patients that require a second procedure and with more advanced atrial disease (greater LA reservoir impairment), but preserved booster function might benefit more from SA, while those with very advanced LA disease (greater LA booster impairment) could potentially be treated just with antiarrhythmic and rate-control drugs. Certainly, further studies on a larger population are required to confirm this recommendation. However, our findings provide data that could generate hypotheses for future research.

One limitation of our study was the relatively small sample size. Only patients in sinus rhythm could be studied, so the effect of ablation and predictors found in this study only relates to this subgroup of patients. Another limitation involves the technique itself, as the platform used was designed for the analysis of the ventricle. Special care in LA tracking must be taken into account; pulmonary veins, and LA appendage could be a problem with tracking, so these structures must be avoided during the delineation of the endocardial border. In our study, we obtained acceptable tracking in all segments in 95% of the patients and our reproducibility was good, as reported elsewhere (20). The translation to other commercially available platforms and vendors of our proposed cut-off values in strain and strain rate measurements requires further validation. Another limitation is that we only performed the analysis in the 4-chamber view; valuable information could also be acquired by adding and averaging LA analysis in the 2-chamber view. Also as chronic hypertension can affect atrial function, the different prevalence of hypertension in the two groups could be a limitation to compare baseline LA function in the CA and SA groups. However, it should not impact on the effect of the intervention in each group.

## Conclusion

The present study proved the efficacy of adding LA function values to LA size to improve the selection of candidates for repeated procedures of AF ablation, whether CA or SA. The analysis of LA function allows a more accurate selection of patients that could suffer an AF recurrence after CA or SA. The surgical procedure does not further impair LA booster function, which must be preserved to avoid dyspnea and improve symptoms in patients with mild hypertrophy or diastolic dysfunction. This could result in improvements in the efficiency of repeated ablation and reduced complications from these procedures. Finally, the clinical impact of these findings in the long-term warrants further investigation.

## Data availability statement

The raw data supporting the conclusions of this article will be made available by the authors, without undue reservation.

## Ethics statement

The studies involving human participants were reviewed and approved by the Comitè Ètic Hospital Clínic de Barcelona. The patients/participants provided their written informed consent to participate in this study.

## Author contributions

All authors listed have made a substantial, direct, and intellectual contribution to the work, and approved it for publication.

## Abbreviations

AF: atrial fibrillation
CA: percutaneous Catheter ablation
LA: left atrial
LASs_*l*_: LA systolic longitudinal strain
LASRa_*l*_: LA late diastolic longitudinal strain rate
SA: Surgical ablation.
